# Cell Culture Model Evolution and Its Impact on Improving Therapy Efficiency in Lung Cancer

**DOI:** 10.3390/cancers15204996

**Published:** 2023-10-15

**Authors:** Viviana Roman, Mirela Mihaila, Nicoleta Radu, Stefania Marineata, Carmen Cristina Diaconu, Marinela Bostan

**Affiliations:** 1 Center of Immunology, Stefan S. Nicolau Institute of Virology, Romanian Academy, 030304 Bucharest, Romania; viviana.roman@virology.ro (V.R.); marinela.bostan@virology.ro (M.B.); 2 Department of Biotechnology, University of Agronomic Sciences and Veterinary Medicine of Bucharest, 011464 Bucharest, Romania; 3 Biotechnology Department, National Institute for Chemistry and Petrochemistry R&D of Bucharest, 060021 Bucharest, Romania; 4 Faculty of Medicine, University of Medicine and Pharmacy Carol Davila, 050471 Bucharest, Romania; stefania.marineata@umfcd.ro; 5 Department of Cellular and Molecular Pathology, Stefan S. Nicolau Institute of Virology, 030304 Bucharest, Romania; directie@virology.ro; 6 Department of Immunology, ‘Victor Babeș’ National Institute of Pathology, 050096 Bucharest, Romania

**Keywords:** 2D and 3D cell culture, organoids, spheroids, tumor microenvironment, tumor-on-chip, lung cancer

## Abstract

**Simple Summary:**

The lack of models capable of reproducing the cellular mechanisms responsible for cancer progression in vivo makes it difficult to develop effective anticancer treatments. In this review, we provide an overview of the progress made in developing 2D to 3D cell culture models and the future challenges associated with microfluidic devices for personalized medicine in cancer therapy, with a particular focus on lung cancer. We also discuss the advantages and limitations of 3D cell culture models, such as spheroids, organoids and bioprinted tissues, as well as microfluidic technology. Finally, we present the challenges and future perspectives of 3D microtechnology in lung cancer and its potential impact on both diagnosis and treatment.

**Abstract:**

Optimizing cell culture conditions is essential to ensure experimental reproducibility. To improve the accuracy of preclinical predictions about the response of tumor cells to different classes of drugs, researchers have used 2D or 3D cell cultures in vitro to mimic the cellular processes occurring in vivo. While 2D cell culture provides valuable information on how therapeutic agents act on tumor cells, it cannot quantify how the tumor microenvironment influences the response to therapy. This review presents the necessary strategies for transitioning from 2D to 3D cell cultures, which have facilitated the rapid evolution of bioengineering techniques, leading to the development of microfluidic technology, including organ-on-chip and tumor-on-chip devices. Additionally, the study aims to highlight the impact of the advent of 3D bioprinting and microfluidic technology and their implications for improving cancer treatment and approaching personalized therapy, especially for lung cancer. Furthermore, implementing microfluidic technology in cancer studies can generate a series of challenges and future perspectives that lead to the discovery of new predictive markers or targets for antitumor treatment.

## 1. Introduction

Lung cancer is one of the most widespread forms of cancer in the world, with high mortality rates and poor prognoses recorded globally [1]. Of the total number of lung cancer cases reported annually, 85% are non-small cell lung cancer (NSCLC), while small cell lung cancers (SCLC) account for approximately 13%, and lung carcinoid tumors only represent 2% [2]. The aggressive progression of lung cancers and the low survival rate associated with poor response to therapy or the appearance of resistance to treatment are due to the rapid metastasis and dissemination of cancer cells to other organs [3]. This invasive process is the final consequence of communication between cancer cells and the surrounding tumor microenvironment [4,5]. Choosing the treatment regimen for lung cancer patients is complicated and depends on factors such as the tumor’s stage and size, its location in the lungs, to which lymph nodes it has spread, and the patient’s age and health. In general, patients with lung cancer are diagnosed late when the tumor is already in an advanced stage, making it challenging to make a favorable treatment decision. Conventional treatments include surgical removal of the tumor, chemotherapy, radiotherapy, or a combined strategy, but the results obtained and the prognosis are generally poor [6].

The limited number of models used in vitro and in vivo for tumor studies hinders the in-depth understanding of cellular behavior and molecular mechanisms responsible for the evolution of tumor processes [7]. In many research laboratories, experiments were performed using primary cells isolated directly from tissue or using well-characterized and stabilized cell lines existing in cell banks such as ATCC (American Type Culture Collection, Manassas, VA, USA) or ECACC (European Collection of Authenticated Cell Cultures, Salisbury, UK) [8,9].

**Figure 1:**
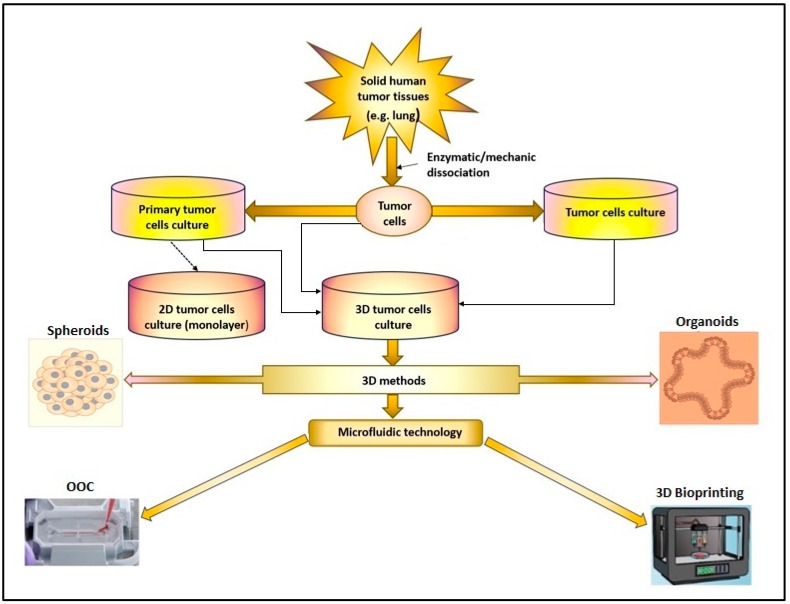
The 2D or 3D cell culture models were developed in research laboratories to study cancer pathologies. (OOC-organ-on-chip).

Primary cell cultures have the advantage of mimicking the genetic characteristics of tumors, facilitating functional experiments. However, obtaining laboratory cultures of tumor cells from tumor tissue can be complex due to the presence of various types of cells [10]. Creating an artificial environment that contains all the necessary components for cell survival and proliferation (nutrients, growth hormones, temperature, pH, and culture) is essential to allow the growth of living cells that are separated from the tumor tissue. Depending on the cancer type, tumor cells can grow in suspension (as in the case of leukemic cells), or they can be adherent (e.g., tumor cells obtained from solid tumors) (Figure 1). In a 2D culture, the expansion and propagation of cells in a controlled manner, cell growth, and division occur in a single plane. However, the major limitation of this primary cell culture is that cells have a finite lifespan [11,12]. Therefore, to ensure the reproducibility of experiments, the alternative option is to use stabilized cell lines. All this information provided by 2D cell cultures opened a new way for 3D culture and microfluidic technology, leading to the creation of highly specialized devices that can imitate in vitro the in vivo conditions more faithfully, creating the necessary premises for the reconstruction of human organs and tissues in vitro [13].

Consequently, this review summarizes the current progress of 2D to 3D cell culture models and future challenges regarding microfluidic devices for personalized medicine in cancer therapy, especially those focused on lung cancer. Finally, we described the future perspectives of 3D microtechnology in lung cancer as well as the impact it can have on diagnosis, surgical approach, and the development of therapeutic protocols [14].

## 2. In Vitro Models for Cancer and Its Research Evolution: From 2D to 3D Cell Cultures

The studies described by Harrison RG [15] show that obtaining and using 2D cell cultures in the laboratory have been known since the beginning of the 20th century. However, after the 1940s, the importance of cell cultures in the research laboratory saw a remarkable evolution, which led to the development of vaccines such as the polio vaccine in 1948. In the years to come, cell cultures were used in the laboratory to obtain as much physiological and biochemical information as possible. This technique proved to be extremely useful in advancing the field of research and allowed scientists to better understand the complex workings of the human body. With continued advancements in technology, it is likely that cell cultures will continue to play a crucial role in scientific discovery and medical breakthroughs [16,17]. Cell cultures and animal models represented a valuable tool for research laboratories and constituted the basis for understanding biological processes at the cellular level and deciphering the mechanisms involved in the pathology of some diseases. In the case of tumor diseases, numerous studies in vitro use 2D cell cultures to identify specific molecules responsible for the disease’s evolution and to analyze the response to conventional therapy [18,19]. To establish the type of radiation treatment or other medical procedures, numerous research studies have been carried out on 2D cell culture models [20,21,22]. Chemotherapy is the main treatment option to destroy lung cancer cells. Many laboratory studies were carried out on 2D cell cultures using tumor cell lines to analyze how cytotoxic drugs act on tumor cells. Thus, it was possible to establish the optimal type and dose of cytotoxic drugs for small cell lung cancer (SCLC) and non-small cell lung cancer (NSCLC). Also, laboratory studies have allowed the deciphering of signaling pathways whose activation or inhibition can influence the sensitivity of tumor cells to chemotherapy [23,24,25]. All this allowed the optimization of radio- and chemotherapy schemes before applying them in clinical trials [26,27,28,29].

In laboratory studies, the use of 2D cultures does not allow the detection of how the cellular or extracellular environment contributes to changes in cellular morphology. Also, it cannot be quantified how tumor cells grow and interact with other cell types or the extracellular environment, processes that can affect the mechanisms involved in the appearance and evolution of tumors. It is possible that through the repeated use of cell lines in vitro, they lose a series of characteristics and no longer present the same molecular heterogeneity as that of the primary tumor cell from which they were obtained. Comparative studies of transcriptomic and/or proteomic profiles of lung cancer patient tumors and cell lines have often demonstrated the presence of divergent data [30,31,32].

In order to better preserve the molecular characteristics of patient tumors, there has been a particular concern of several groups of researchers who have attempted to obtain lung tumor cell cultures derived from patient biopsy samples. Although this is a goal, the success rate in obtaining and using tumor cell cultures from lung biopsies is less than 50% and is associated with high financial costs [33,34,35].

Using cell lines or primary cell cultures obtained from tumor tissue and animal studies has provided considerable information on the biology and pathology of various diseases and facilitated the testing of different drug effects (Figure 1). However, the limitations of these study methods must be accepted regarding the impossibility of accurately reproducing the conditions of the cellular microenvironment existing in vivo, as well as numerous ethical aspects. Also, the results of laboratory studies on animal models or 2D cell cultures on the efficacy of new drugs in treating various diseases have often been insufficient to allow their use in clinical trials [36]. In addition, in vivo experiments are expensive, require a long study time, and involve the sacrifice of the animals used in the study. Therefore, the emergence and development of new research models such as spheroids, organoid, or microfluidic platforms that mimic the existing in vivo conditions have contributed to the reduction of the number of animals used in the laboratory or to the replacement of animal studies where possible [37,38]. All this has contributed to the emergence and development of research models that will enable the replacement of laboratory studies on animals with studies on 3D structures performed in vitro, but which ensure a functionality similar to that existing in vivo (Figure 1).

## 3. 3D Cell Culture Model Revolution: Spheroids and Organoids

To mimic the existing in vivo situation as reproducibly as possible, informations about cell–cell relationships, cell–extracellular matrix (ECM) interactions, and potential connections with various other cell types were used. Numerous studies on how to obtain 3D cultures were carried out and published between 1975 and 1990. The first 3D cultures that attempted to define three-dimensional tissues in vitro using human progenitor cells were described in 1975 by Rheinwatd and Green [39]. Then, in 1987, Zimmermann [40] obtained in vitro the 3D cultures from lung tissue, and at the beginning of the 1990s, Hachitanda and Tsuneyoshi [41,42] obtained the 3D cultures derived from neuroblastomas. One of the first studies to present techniques for obtaining 3D cultures and their advantages for pharmacological studies is Mina Bissell’s 1980 paper [43].

An essential step in developing new types of cell cultures was using a biocompatible medium/matrix to ensure optimal cell growth conditions so that their integrity would not be affected during testing. The development of 3D cell culture models was supported by the performance of technology that made it possible to evaluate the complexity of cell behavior in tissues and organs, offering a much larger scope of information than that obtained in cells grown in a 2D culture model [44,45]. The evolution from creating the first 3D cultures to generating spheroids and organoids was high-speed after the 2000s.

The three-dimensional architecture is generated in vitro, by culturing or co-cultivating different types of cells existing in the target tissue/organ structure, with the help of so-called scaffolds that function as matrices to ensure 3D morphological assembly. The three-dimensional architecture is generated in vitro, by culturing or co-cultivating different types of cells existing in the target tissue/organ structure, with the help of so-called scaffolds that function as matrices to ensure 3D morphological assembly.

**Figure 2:**
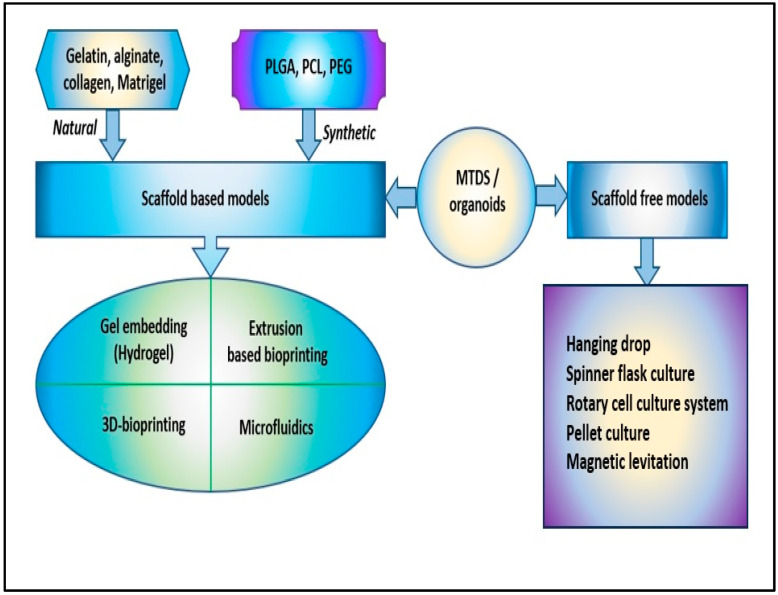
The scaffold-based and scaffold-free models to obtain 3D cellular structures, such as multicellular tumor-derived spheroids (MTDS) and organoids [poly(lactic-co-glycolic) acid (PLGA), polycaprolactone (PCL), and polyethylene glycol (PEG)].

In the development of 3D cell culture systems that mimic as reproducibly as possible the physiological processes that take place in vivo, an important role was played by the types of technologies used, starting from multicellular spheroids, hydrogels, anchorage and scaffold approaches, 3D bioprinting, hanging drop microplates, up to microfluidics/lab-on-chip technologies. In the processes of generating spheroids and organoids, the use of scaffolds played an essential role because they provide a supporting structure for cells to grow and differentiate in three dimensions, which in turn helps to better mimic the in vivo microenvironment and advance our understanding of cellular biology [46]. For spheroids, scaffolds can be made of various materials, including hydrogels, nanoparticles, and microfabricated devices, and they can be designed to mimic the mechanical, chemical, and biological properties of the in vivo microenvironment. By providing a supportive structure, scaffolds allow cells to grow three-dimensionally and regulate cellular behavior, such as cell migration, proliferation, and differentiation (Figure 2) [47].

In the creation of organoids, scaffolds are often used to mimic the structural and mechanical properties of the organ of interest and to provide a supportive environment for organ-specific stem cells or progenitor cells to differentiate into functional organ-like structures. Using scaffolds in organoid culture allows researchers to better understand the complex interactions between cells in a three-dimensional context and to develop functional organ-like structures in the laboratory.

Hydrogels are polymers of covalently or noncovalently linked molecules; they show similarities with the natural extracellular matrix and are of great interest for realizing 3D cell cultures [48,49]. Initially, the more compatible with 3D cell culturing were hydrogels derived from collagen, matrigel, and alginate. However, their complex and rigid structures did not allow the design of 3D models with the characteristics necessary for studies at the cellular level. This has led to the design of synthetic hydrogels, a series of cross-linked polymer chains used to encapsulate cells, providing an ideal 3D growth environment. Hydrogels based on poly(ethylene glycol) or polyacrylamide can be easily controlled by changing the density of the polymer and the molecular weight and allowing the crosslinking conditions that ensure the efficient encapsulation of proteins and cells to be maintained [50,51]. The reduced costs for obtaining hydrogels allowed the synthesis of several types of hydrogels based on polyethylene oxide (PEO), poly(methacrylic acid) (PMMA), polypropylene fumarate-co-ethylene glycol (P(PF-co-EG)), poly (acrylamide) (PAAm), and poly N-isopropyl acrylamide (PNIPAAm) (Figure 2) [52,53].

3D cell culture models provide information about cell structure, tumor microenvironment, and functional processes, such as cell differentiation and proliferation. Also, 3D cell cultures enable the efficient study of intercellular interactions and factors responsible for the evolution of tumor processes and allow the analysis of various changes in cellular biochemistry that can be easily detected, including changes in mRNA splicing and gene expression [54].

In addition to the valuable information that can be obtained with the help of 3D cell cultures regarding the pathophysiology of each tumor process, very important studies analyze the toxicity of potential therapeutic agents before they are introduced into clinical trials.

Over the past two decades, 3D cell cultures have been developed by interlinking multidisciplinary insights from biology, electronics, informatics, and medicine. The methods used to obtain 3D cellular structures, such as spheroids and organoids, have been classified into scaffold-based and scaffold-free systems (Figure 2) [55].

### 3.1. Spheroids

Tumor spheroids are spherical cell units obtained by performing a 3D cell culture with or without scaffolds. Spheroids are obtained starting from unicellular suspensions that, under certain conditions, interact with the nearby cells and create aggregates that float freely. The principle of these methods is based on the process of cell aggregation with the help of some molecules (integrins and extracellular matrix proteins) involved in creating desmosomes and cellular junctions responsible for intercellular interactions [56]. Different scaffolds (hydrogels, nanoparticles) can be used to imitate the mechanical, chemical, and biological properties of the microenvironment existing in vivo to obtain spheroids [57]. These scaffolds function as support structures that ensure the growth of cells in a three-dimensional manner and allow the possibility to regulate processes such as cell migration, proliferation, and differentiation.

Several scaffold-based models for obtaining tumor spheroids have been described, such as extrusion-based bioprinting, gel embedding, or microfluidic methods.

Extrusion-based bioprinting is a 3D printing technique that creates complex structures, including spheroids, from bio-ink composed of living cells and a hydrogel matrix. The specific protocols and conditions can vary depending on the type of cells, hydrogel, and bioprinter used.

*Gel embedding* needs a 3D platform for cells to grow on and interact with. The first stage involves the cell culture and gel matrix realization. The cells and the gel matrix are transferred to a sterile culture dish and placed in an incubator with the appropriate environmental conditions (temperature, humidity, and CO_2_ levels). The spheroids are maintained in culture by changing the medium according to experimental requirements and monitoring spheroid formation and growth using microscopy.

*Microfluidic methods* ensure precise control over the size, composition, and environmental conditions in obtaining spheroids. The first step is to choose and purchase a suitable microfluidic device for spheroid generation. The designs of existing microfluidic devices can be of different types: droplet generators, flow-focusing channels, or microwell arrays. Manufacturing methods may include soft lithography, micro-milling, or 3D printing. Microscopy or other analytical techniques involving the incorporation of microsensors are used to monitor the growth and behavior of the spheroid over time.

Also, there are scaffold-free models such as pellet culture systems, hanging drop culture, magnetic levitation, rotary cell culture, and spinner flask culture.

*Pellet culture system* is used to create concentrated 3D cell pellets (spherical aggregates) that are obtained by gently detaching from the walls of tubes (a spheroid tube) after cell suspensions at various concentrations have been centrifuged and incubated overnight at 37 °C, 5% CO_2_ atmosphere.

*Hanging drop culture* is a technique used for the formation of 3D cell aggregates or spheroids. It involves placing a small droplet of cell suspension (usually containing cells and a culture medium) on the underside of a culture dish lid. Due to gravity, the droplet hangs down from the lid, and cells within the droplet aggregate together in a 3D structure over time. This method is particularly useful for studying cell–cell interactions and differentiation [58,59].

*Magnetic levitation method*—nanoparticles and cells were cultured together and kept in a magnetic field until cell spheroids formed were detached from the dish bottom and manipulated with the help of a magnet [60].

*Rotary cell culture system (RCCS)*—single-cell suspensions were placed in the rotating chamber at an initial speed of 12 rpm and were placed inside a humidified 37 °C, 5% CO_2_ incubator. This rotator system (Synthecon Inc., Houston, TX, USA) is connected to external power supplies to the incubator. All procedures were performed in sterile conditions under a laminar flow hood, and tumor spheroids were obtained in around 15 days [61].

*Spinner flask culture* is a method used for the cultivation of cells in suspension. It typically involves a flask equipped with a magnetic stir bar (or similar agitation mechanism) that keeps the culture medium and cells in suspension. The agitation provided by the spinner flask ensures even distribution of nutrients and oxygen to the cells and prevents settling. It is commonly used for scaling up the production of cells, such as in bioprocessing and bioreactor systems [62].

Different forms of multicellular spheroids are obtained depending on the type and density of tumor cells and the methods used. Multicellular tumor spheroids (MTS) are mainly generated from tumor cell lines. These cells grow in spherical colonies in suspension and reproduce tumors’ proliferative and metabolic activity. These spheroid models are relatively easy to obtain and maintain and, thus, facilitate the realization of testing studies of new drugs [63,64]. Multicellular tumor-derived spheroids (MTDS), also called tumorspheres, are prepared from the mechanical or enzymatic dissociation of tumor tissue into a single-cell suspension [65].

Although obtained by the same methods used for MTS, tumor spheres (MTDS), being a single-cell suspension of tumor tissue, are characterized by the abundance of many cancer stem cells (CSCs), which can be highlighted by analyzing the expression of specific markers such as CD133, CD44, and ALDH. In addition, CSCs have a role in the differentiation process into tumor tissue-specific cell lines. Thus, the enrichment of MTDS cultures with CSCs facilitates their use for deciphering tumor formation in vivo [66,67]. The studies carried out with the help of MTDSs allowed the investigation of the response to chemotherapy and especially the establishment of the process of resistance to therapy and the appearance of tumor metastasis, which seems to be associated with CSCs [68].

Heterotypic spheroids were obtained using 3D modeling methods by coculturing multiple cell types such as tumor cells, stromal, and immune cells. Using heterotypic spheroids in research studies tries to recreate the communications between tumor, stromal, and immune cells similar to the signaling network in vivo solid tumors [69].

Despite this information, spheroids are simple, spherical aggregates of cells with no distinct tissue layers or specialized structures. Spheroids are often used for basic research, drug screening, and toxicity testing. They are less specialized and may not accurately recapitulate the function of specific organs [70,71].

Lung cancer is a more aggressive disease characterized by high clonal and morphological heterogeneity of tumors. The complex multicellular tumor microenvironment characteristic of lung cancer influences essential biological processes that define tumor growth, metastasis, response and resistance to therapy. Various studies describe the methods used to obtain spheroids and their role in deciphering the pathological processes pertaining to lung cancer [72,73,74]. Tumor-derived spheroid models allowed the study of heterogeneous cell–cell or cell–ECM interactions and the evaluation of the role of oncogenes or the immune system in the tumorigenesis of lung cancers [75,76]. Spheroid models are widely used in research for lung cancer due to their ability to mimic the in vivo microenvironment of tumors. Some specific applications of spheroid models in lung cancer research studies are shown in Table 1.

Spheroid models have revolutionized lung cancer research by offering a more physiologically relevant platform for studying the disease. They have been instrumental in drug discovery, resistance studies, metastasis research, and personalized medicine approaches, ultimately facilitating the development of new and more effective lung anticancer strategies [82,83].

For example, spheroid models have been used to test the response of lung cancer cells with specific mutations (e.g., EGFR mutations) to targeted drugs like EGFR inhibitors (e.g., erlotinib) [84,85]. Spheroid models have helped assess the potential of immunotherapies for lung cancer. They allow researchers to study interactions between lung cancer cells and immune cells within a 3D environment, providing insights into the efficacy of immune checkpoint inhibitors (e.g., pembrolizumab) and other immunotherapeutic approaches. Researchers have used spheroids to explore the synergistic effects of combining different treatment modalities. For instance, they can assess the combination of chemotherapy with radiation therapy or immunotherapy to determine if these approaches work better together than individually.

**Figure 3:**
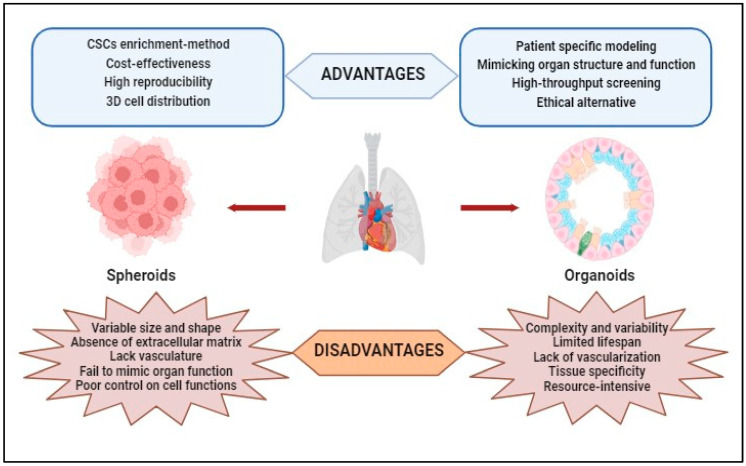
Advantages and disadvantages of using spheroids and organoids obtained through 3D techniques in laboratory studies.

The literature mentions that the spheroidal models have certain limitations caused both by the lack of control over the formation of hetero-spheroids (structures with a different architecture than that existing in the solid tumor can be generated) and by the difficulty of obtaining consistent and reproducible results because the spheroids obtained under identical conditions vary both in shape and size [86]. Also, spheroid models can be more expensive and difficult to maintain than traditional 2D cell cultures due to the need for specialized equipment and reagents. Additionally, not all cell types can form spheroids or maintain cell functions in a 3D culture model [87]. The advantages and disadvantages of using spheroids in laboratory experiments are shown in Figure 3.

Despite these limitations, spheroid models are valuable tools in medical research, particularly for tumor biology studies and drug testing. The more realistic microenvironment provided by spheroids can lead to more accurate results and a better understanding of disease processes than 2D cell cultures.

### 3.2. Organoids

Studies on organ physiology have provided clues to organogenesis and allowed the development of in vitro tissue growth techniques, leading to 3D organoids in vitro [88,89]. Organoids are 3D structures that reproduce tissue-specific characteristics from the distribution of cell types to the structural organization and function specific to the analyzed tissue [90]. The size of organoids can vary depending on the specific type of organoid and the methods used to culture them. Organoids are typically smaller than actual organs but larger than traditional cell cultures. The size of an organoid is influenced by several factors, including the type of tissue or organ being modeled, the starting material (e.g., stem cells or tissue fragments), and the culture conditions [91].

Scaffolds can be used in the formulation of organoids, but their use depends on several factors, including the type of tissue or organoid being developed and the specific goals of the research or application. Scaffolds are three-dimensional structures or materials that provide physical support for cells to grow and organize into tissue-like structures. Scaffolds can replicate a tissue’s natural extracellular matrix (ECM), providing a substrate that encourages cell adhesion, migration, and differentiation. This is particularly useful for certain types of organoids that rely on cell–cell and cell–ECM interactions to develop properly [92]. Also, scaffolds can help guide the organization of cells within the organoid, leading to a more organized and functional tissue structure. However, not all organoid cultures require scaffolds. Some types of organoids, particularly those derived from stem cells, can self-organize and form tissue-like structures without the need for a scaffold.

In summary, organoids aim to replicate the structural and functional features of a specific organ or tissue. They often exhibit organized tissue architecture, including epithelial layers, lumens, and sometimes even vascularization, resembling the organ they model. Organoids are typically generated through more complex and specialized protocols involving differentiated stem cells or progenitor cells in 3D culture systems. These protocols often require specific growth factors and culture conditions to guide organoid development. The development of organoids was made possible by advances in stem cell biology, tissue engineering, and imaging technologies that allowed the study of cellular behavior in three dimensions. Combining these advanced techniques allowed researchers to better understand complex cell interactions and create functional organ-like structures in the laboratory [93].

Over time, organoids have become increasingly sophisticated and are now very useful in studying a variety of organs, including the gut, liver, pancreas, and brain. They have also become useful tools for studying disease and drug discovery and provide a more realistic representation of cellular interactions and functions than traditional 2D cell cultures [94,95].

For example, the organoids obtained from patients with specific genetic mutations, such as EGFR mutations or ALK rearrangements, were used to test the efficacy of targeted therapies, such as tyrosine kinase inhibitors, and evaluate the potential for drug resistance development [96]. Using organoids was possible to investigate the response to immunotherapies like immune checkpoint inhibitors, assess the immune cell infiltration, and study interactions between cancer cells and the immune system [97,98]. Also, lung cancer organoids provide a platform to study the heterogeneity of tumors and the role of cancer stem cells [99].

For research studies, 3D organoids have several *advantages*: (i) Patient-Specific Modeling—Organoids can be derived from patient-specific induced pluripotent stem cells (iPSCs), allowing researchers to create disease models that closely resemble a specific individual’s biology; (ii) Mimicking Organ Structure and Function—3D organoids better replicate the complex three-dimensional architecture of real organs, allowing researchers to study cell–cell interactions, tissue organization, and organ-specific functions more accurately; (iii) High-Throughput Screening—Advances in automation and miniaturization techniques have enabled high-throughput screening of drugs and compounds using organoids, making drug discovery more efficient; and (iv) they provide a more ethical alternative to animal testing or human trials (Figure 3).

3D organoid models are constantly subject to improvement from one study to another. Currently, some disadvantages can be pointed out in order to find more viable solutions.

3D organoid models also present some *disadvantages*: (i) Complexity and Variability—organoid cultures are complex and heterogeneous, which can make them challenging to work with and analyze; (ii) Limited Lifespan—organoids have a finite lifespan in culture, making it difficult to study long-term processes or conduct extended experiments; (iii) Lack of Vascularization—many organoids lack a functional vascular system, limiting their ability to replicate the complex interactions between tissues and blood vessels found in vivo; (iv) Tissue Specificity—organoids may not fully represent the diversity of cell types and tissue structures found in actual organs. Some complexity may be lost or oversimplified in the culture process; and (v) Resource-Intensive—establishing and maintaining organoid cultures can be resource-intensive and time-consuming (Figure 3).

The 3D organoid models provide a valuable tool for studying lung cancer and advancing our understanding of this complex disease. The applications of organoid models in cancer research are shown in Table 2.

### 3.3. 3D Bioprinting Model: Evolution and Application in Medicine

The technological advances registered by tissue engineering and regenerative medicine associated with biofabrication and additive manufacturing have allowed the development of 3D bioprinting [106]. Creating a 3D bioprinting model started with the general notions of the architecture of tissue, and then the information was introduced about the types of component cells in the extracellular matrix (ECM), as well as the nature of biochemical signals and the role of growth factors necessary for the realization of physiological functions [107]. The type of bioreactor used plays a crucial role in maintaining the microenvironmental conditions and regulating specific mechanical stimulations [108]. Finally, with the help of computers, it was possible to generate programs and software that can control the generation of biomimetic tissue at the nano or micro level, corresponding with great accuracy to the existing tissue in vivo [109].

In cancer research, 3D bioprinting is a rapidly developing field in which cells, tissues and even organs can be created using a printer [110]. In summary, 3D bioprinting technology involves creating three-dimensional structures by layering bio-ink, a material composed of living cells and other biological materials, in a precise and controlled manner.

**Figure 4:**
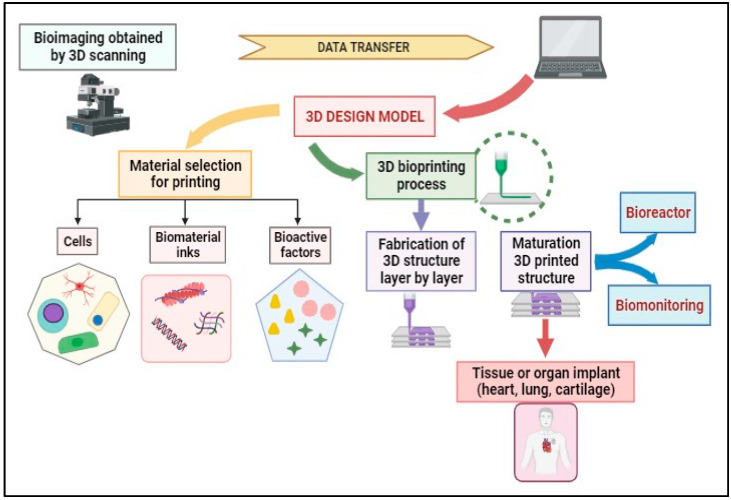
3D bioprinting process flow.

The 3D bioprinting process can be achieved via distinct steps that are presented in Figure 4. Magnetic Resonance Imaging (MRI) and Computed Tomography (CT) are medical imaging techniques used to visualize the internal structures of the human body. While they have similarities, they operate on different principles and provide complementary information. The scanning results from MRI and CT contribute to creating 3D models, which allow the choice of materials (cells, bioactive factors, and bio-inks) and the selection of the printer. The second step involves placing the bio-ink in the printer cartridge and forming the biological constructs on a scaffold layer-by-layer according to the prepared digital model to obtain a 3D structure. The post-bioprinting phase aims to preserve the biological structure obtained and supply the required physical or chemical signals for tissue reorganization and growth.

To achieve 3D bioprinting, the essential factors are surface resolution, cell viability, and biological materials used for printing [111,112]. There are several 3D Bioprinting models. Inkjet-based bioprinting functions similarly to a regular inkjet printer, where droplets of bio-ink are ejected from a printhead onto a substrate [113]. Extrusion-based bioprinting technique creates complex structures from bio-ink composed of living cells and a hydrogel matrix [114]. Laser-based bioprinting uses a laser to selectively target and transfer cells or biomaterials from a donor substrate to a receiving substrate [115]. Stereolithography-based bioprinting uses a laser or UV light to selectively polymerize layers of a photopolymerizable bio-ink, solidifying it one layer at a time [116].

One of the main applications of 3D bioprinting in cancer research is creating in vitro tumor models. Researchers can create a precise replica of the original tumor by printing cells isolated from a patient’s tumor. This allows them to study cancer in a controlled environment and test the effectiveness of different treatments, such as chemotherapy or immunotherapy, in a way that closely mimics the in vivo situation [117,118].

Another application of 3D bioprinting in cancer research is the creation of personalized cancer vaccines. By 3D bioprinting using a patient’s tumor cells mixed with immune-stimulating agents, researchers can create a vaccine tailored to the patient’s cancer. This can significantly improve the effectiveness of cancer vaccines and provide a personalized treatment approach [119].

3D bioprinting can revolutionize how the interrelationships between cancer cells and the surrounding tissue can be analyzed, leading to a better understanding of the mechanisms underlying tumor growth and invasion. It can also create tissues and organs for transplantation or test new drugs’ effectiveness. Although it is a relatively new technology, it has shown great promise in medicine and can be used to develop areas of interest such as Tissue Engineering, Drug Testing, Personalized Medicine, Disease Modeling, Education, and Training.

### 3.4. 3D bioprinting and Its Applications in Lung Cancer

3D bioprinting is a cutting-edge technology that allows the creation of three-dimensional structures by layering materials such as cells, proteins and biomaterials [120,121]. The mechanical, structural, and biological complexity of bioprinting technologies has made it possible to approach studies on tissues from vital organs such as the liver, pancreas, lung, heart, or skin [122,123].

The lungs are organs with complex structures and compositions that ensure breathing. In addition, more than 60 types of cells performing other functions have been described in the lung and alveolar parenchyma. The human lung is made up of two areas: the conducting area of the lung comprising the trachea, main bronchi, and terminal bronchioles, and the respiratory region comprising the respiratory bronchioles, alveoli, and alveolar sacs. The complicated architecture of the lung is associated with complex lung function because the pulmonary veins supply the respiratory zone with deoxygenated blood, and the bronchial arteries provide the more metabolically active conductive site with oxygenated blood [124]. However, with the help of 3D bioprinting, it is currently possible to manufacture various lung tissue segments [125,126], but the complete network of the airways from the trachea to the terminal alveolar sacs has yet to be realized.

In this context, in vitro studies are difficult to achieve, and this has led to significant challenges for researchers studying the appearance and evolution of lung tumors to understand the physiological processes that take place in vivo [127].

The use of 3D bioprinting in the case of lung tissue was difficult to approach, on the one hand, due to the complex structure of the lungs and on the other hand, due to the lack of bio-inks that would ensure all the necessary conditions for the generation of lung tissue [128,129]. However, in recent years, synthetic and composite polymer hydrogels and micro and nanostructured biomaterial inks have appeared and developed, favoring lung tissue design and 3D bioprinting [130,131].

In addition, using these “smart” techniques allows the realization of the 3D bioprinting lung tissue matrix and ensures the monitoring of the cellular response to different biological signals.

Initially, 3D bioprinting models were designed using cell lines that contributed to selecting and analyzing the effectiveness of anti-tumor therapy specific to each type of cancer studied. For example, in the case of lung cancer, there are several studies in which standardized tumor cell lines such as A549 or 95-D lung cancer cells were used to create a tumor-like lung cancer model with the help of 3D bioprinting. In this regard, the tumor cells were dispersed into a hydrogel of the alginate–gelatin bio-ink type, and it was observed that cell viability was not affected during the printing process [132]. However, studies using 3D bioprinted models have demonstrated that the response of tumor cells to therapy can be influenced by the interaction with other types of cells that belong to the stroma or to the immune system. Therefore, currently, they are trying to include tumor cells isolated from the patient to create 3D culture models as faithfully as possible to the model existing in vivo for the most effective testing of antitumor drugs [133].

**Figure 5:**
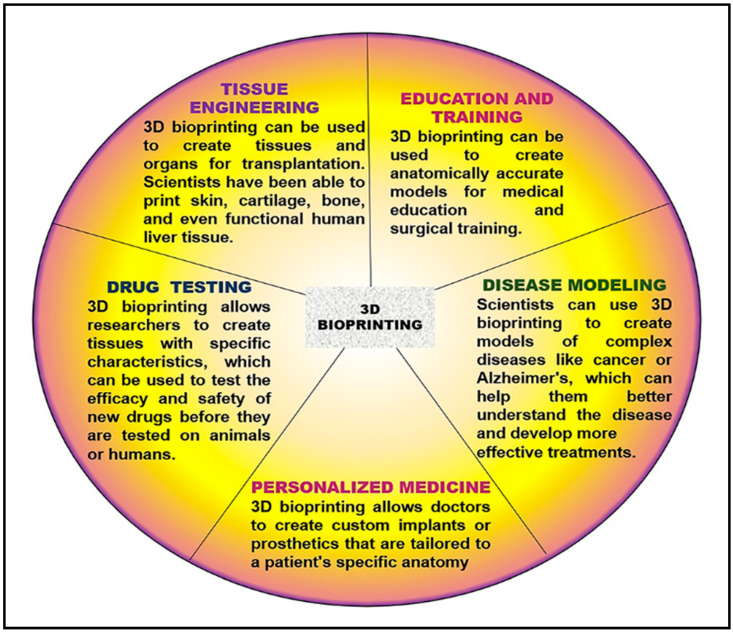
3D Bioprinting applicability in medicine.

In the field of cancer therapy, 3D bioprinting offers a new approach to the treatment of lung cancer. 3D models of lung tumors obtained by bioprinting technology can be used to study the behavior of cancer cells and to test the effectiveness of various drugs [134,135]. Also, this technology can be used to print drug-loaded nanoparticles that can be delivered to the tumor site, improving the efficacy of the therapy and reducing the side effects [136,137]. Remarkably, tissue engineering shows that 3D bioprinting can create lung tissue for transplantation. This technology can be used to print functional lung tissue that can be used to replace damaged tissue in lung cancer patients [138]. Overall, 3D bioprinting offers promising opportunities to treat lung cancer, and further research is needed to explore its full potential in this field (Figure 5).

However, 3D bioprinting models used in vitro cannot be successfully used for drug screening because the vascularization networks of the obtained tissues or organs are not made [139]. To overcome these limitations, the integration of 3D bioprinting with microfluidics was attempted, which enabled the fabrication of vascular channels [140]. The advancement of 3D bioprinting techniques has facilitated the growth and progress of microfluidic technology [141,142].

### 3.5. Microfluidic Technology Concept and Its Impact on Improving Cancer Therapy

Microfluidic technology, or lab-on-a-chip technology, involves manipulating and controlling small amounts of fluids (typically in the microliter or nanoliter range) within miniaturized devices called microfluidic chips. These chips contain channels, chambers, and other components that enable precise handling, analysis, and manipulation of fluids on the microscale. These devices can be made from materials such as glass, silicon, or polymers and can be designed to perform a variety of functions. Microfluidic technology is used to create both organ-on-a-chip and tumor-on-a-chip systems.

**Figure 6:**
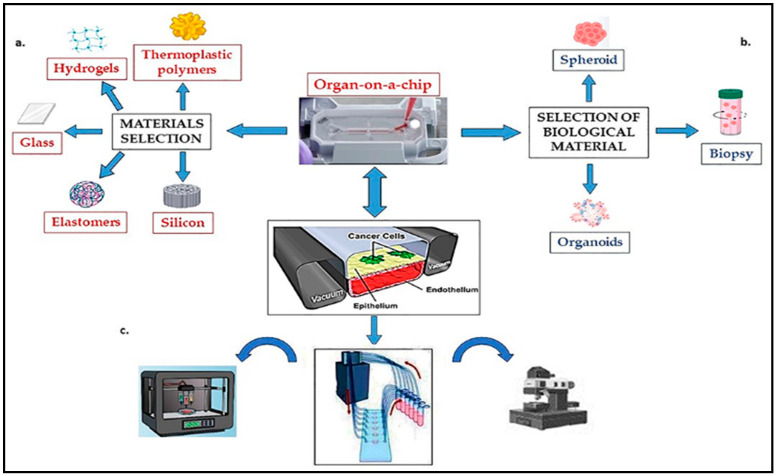
Components of functional tumor-on-a-chip system: (**a**) material selection and microchip fabrication; (**b**) selection of biological material; (**c**) peripheral equipment (Incubators, Pumps for perfusion, Mechanical stimulation, Controls and sensors, Microscope).

*Organ-on-chip* (OOC) platforms utilize microfluidic technology to recreate the structure and function of human organs in vitro. These devices contain microchannels lined with living cells that mimic the complex physiology of organs, allowing for detailed studies of organ responses to drugs and other stimuli (Figure 6).

OOC systems have the potential to replace animal models in drug testing and provide insights into organ-level responses in a more human-relevant manner. Those devices permit studying human physiology and disease more realistically and accurately than traditional cell cultures or animal models. Some OOC systems are currently used in research and medicine (Table 3).

Microfluidics is a technology that allows for the development of highly advanced in vitro tumor models. These models closely resemble the physiological and structural characteristics of tumors found in the human body. So, tumor-on-chips are used to study tumor biology and gain insights into tumor behavior, which can ultimately lead to more effective therapies [156]. The tumor-on-chip creates a system in vitro replicating key aspects of the in vivo tumor microenvironment, including the mechanical forces, fluid flow, and nutrient gradients the cells experience in the body. Tumors are complex environments, including various cell types, involving tumor, immune, and stromal cells. By incorporating multiple cell types into tumor-on-chips, researchers can more accurately replicate the complexity of the in vivo tumor microenvironment and improve the accuracy of their results. All of these allow researchers to study the behavior of cancer cells in a more realistic and controlled situation, which can provide new insights into tumor growth and other important aspects of cancer biology. In medicine, tumor-on-chips can be used to grow and study tumor cells, and researchers use microscale tumor models to analyze blood or tissue samples for cancer-associated biomarkers or cancer-related gene mutations [157,158]. Tumor-on-chips have a multitude of potential applications, including drug discovery, optimizing time-and-dose drug delivery, assessing the effectiveness of other delivery methods, developing point-of-care diagnostics, and offering the possibility of approaching personalized medicine to be approached more easily [159].

Microfluidic technology has several advantages over traditional methods in medicine, such as improved precision, reduced sample sizes, and increased speed and automation. In medicine, microfluidic technology promises to revolutionize, especially the field of oncology, by providing real information regarding the tumor microenvironment and the tumor structure [160]. This technology ensures the creation of microfluidic chips, devices that can provide a better understanding of the behavior of tumor cells and how they respond to different types of treatment. In addition, advanced imaging techniques, such as live cell imaging, can be used to monitor the response of cancer cells to therapy in real time. Using these tools and techniques, researchers can provide new insights into how cancer cells respond to therapy and optimize treatment strategies.

Microfluidic technology offers unique opportunities for various diagnostic applications, including infectious disease detection, biomarker monitoring, or the possibility of combining with next-generation sequencing as futuristic genetic analysis [161]. This technology has the advantage that all analytical steps, such as sample preparation, mixing, and detection, are integrated into a single platform. Such devices play a crucial role in the development of liquid biopsy techniques, which involve the analysis of tumor-derived components from body fluids such as blood or urine. The devices can efficiently capture and isolate circulating tumor cells (CTCs), exosomes, or cell-free DNA from these samples, providing valuable information about tumor progression and treatment response [162,163]. Through this technology, we can create miniaturized, biomimetic environments that mimic the complex conditions in the human body. Microfluidic chips enable the simultaneous testing of multiple drug candidates, accelerating the discovery and optimization of new cancer therapies. Furthermore, by incorporating microscale channels, valves, and pumps, microfluidic devices can precisely control drug release by directly modulating the flow rates, composition, and size of the delivery system at tumor sites. This technology enables the targeted delivery of chemotherapy drugs directly into the tumor, improving drug efficacy, minimizing systemic toxicity, and reducing side effects [164]. These technologies can process small sample volumes, making them ideal for analyzing limited biological material such as circulating tumor cells or rare cell populations [165].

Microfluidic technology is a rapidly evolving field that aims to recreate the functions and physiology of human organs on a microscale device. While it holds immense potential for various applications, it also has advantages and disadvantages (Figure 6).

This technology presents numerous advantages because it provides a more accurate representation of human organs than traditional in vitro cell cultures or animal models (mimicking the microenvironment, cellular interactions, and physiological responses of human organs). It also enables the simultaneous testing of multiple compounds or treatments and provides valuable screening and rapid evaluation of drug candidates, reducing the costs and time associated with traditional drug development processes.

The technology offers an ethical alternative for testing drug efficacy and toxicity, thus minimizing the need for animal experimentation. A significant advantage is that microfluidic chips can be customized to reproduce specific patient conditions or genetic profiles, and it opens up possibilities for personalized medicine. Being a state-of-the-art technology, it incorporates sensors and imaging techniques for real-time monitoring of cellular responses, biochemical markers, and physiological parameters. All this ensures the obtaining of valuable data about the effects of drugs, the disease’s progression, and the tissues’ functionality, improving their understanding of the physiology of the organs.

However, several disadvantages of microfluidic technology must be mentioned: the high complexity of designing and manufacturing devices with precise cellular arrangements and functional integration. Last but not least, the high cost and the technical challenges of developing and maintaining chip systems can limit their widespread adoption.

This technology has limitations in mimicking human organs’ full complexity and heterogeneity (all the cell types, vasculature, and intricate organ architecture). In addition, standardization and validation protocols are still being developed.

While microfluidic technology has several advantages, there are still hurdles to overcome before it becomes a routine tool in research and clinical settings. However, with ongoing advancements and interdisciplinary efforts, these limitations can be addressed, leading to more widespread adoption of these technology systems in the future.

Although *microfluidic technology and 3D bioprinting* are both cutting-edge technologies with applications in both biology and medicine, a comparative analysis highlights some significant differences. Thus, microfluidic technology primarily controls and manipulates fluidic systems at the microscale, while 3D bioprinting creates three-dimensional structures composed of living cells and other biological materials. Microfluidic devices can also be made from various materials, including plastic, glass, and silicon, while 3D bioprinting typically uses hydrogels, cell suspensions, and other biocompatible materials to create the printed structures. Microfluidic devices are extremely precise and can be used to control and manipulate fluids with great precision, while 3D bioprinting is still a developing technology that requires further refinement to achieve the precision required for some applications. The scope of applicability of microfluidic technology encompasses a variety of fields, including biochemistry, cell biology, and drug discovery, while 3D bioprinting is mainly used in the fields of regenerative medicine and tissue engineering.

In the context of cancer research, microfluidic technology and 3D bioprinting are still in their early stages and can potentially revolutionize how we approach medical treatments and procedures in cancer.

### 3.6. Role of Tumor Lung-on-Chip to Improve Cancer Therapy

OOC technology has become a promising tool for studying lung cancer. This technology involves using microfluidic devices containing tiny 3D structures that mimic human organs’ physical and functional properties. In the case of lung cancer, tumor organ chips can be used to study the behavior of lung cancer cells in a controlled and reproducible environment [166]. The development of tumor lung-on-chip technology involves a microfluidic platform that recreates the complex microenvironment of a tumor in a laboratory setting. It typically involves culturing cancer cells on a chip, which contains channels or compartments that mimic the structure and function of blood vessels, extracellular matrix, and surrounding tissues. This technology allows researchers to study tumor growth, invasion, and response to various treatments in a more controlled and representative environment than traditional cell culture methods [167].

For example, tumor lung-on-chip devices can be obtained using 3D printing technology such as lithography. These techniques allow for the precise and reproducible fabrication of microfluidic channels and chambers that mimic the lung structure [168]. Advances in cell culture techniques, such as induced pluripotent stem cells (iPSCs), have made it possible to generate lung cells that can be used to model the human lung in vitro. These devices often incorporate sensors and imaging techniques to monitor the response of lung cells and tissues to different stimuli. For example, fluorescent imaging can track the movement of cells and molecules within the device. Also, electrical sensors can measure lung cells’ response to mechanical and chemical stimuli [169].

Tumor lung-on-chip devices rely on microfluidic technology to simulate the flow of air and fluids through the lung cells. Microfluidic channels and valves are used to control the airflow and fluids through the device and ensure the study of the response of lung cells and tissues to different environmental conditions [170].

Tumor lung-on-chip represents a relevant model for studying lung physiology. Until now, two models of the human lung have been created—the Alveolus Lung-Chip and the Airway Lung-Chip—which facilitate the study of pulmonary physiopathology and the drug’s effect. After loading the tumor-on-chips with the biological materials, they are connected in a module responsible for the nutrient media distribution and the control of the dose of the compound. The entire system is controlled by software that allows users to design tumor-on-chip studies, monitor the process remotely, and analyze the generated results (Figure 6). Tumor lung-on-chip can be used to test the efficacy of new drugs and assess their toxicity in a more physiologically relevant setting. This can provide valuable information for the development of new treatments for lung cancer. In addition, using tumor lung-on-chips, the interactions between lung cancer cells and other cells and tissues in the microenvironment can be studied. This can provide essential insights into lung cancer progression mechanisms and may help identify new therapy targets. There have been several studies utilizing tumor lung-on-chip models to study lung cancer. For example, researchers have developed a tumor lung-on-chip model that recapitulates the tumor microenvironment and allows for the testing of various cancer treatments [171]. This model utilizes human lung cancer cells, stromal cells, and extracellular matrix components to simulate the tumor microenvironment. It has been used to evaluate the effectiveness of different cancer drugs, including chemotherapeutic agents and targeted therapies [172,173,174]. Another example is a study that developed a tumor lung-on-chip model to study the interactions between tumor cells and immune cells in the tumor microenvironment. This model allowed analysis of the dynamics with which immune cells can infiltrate the tumor site and how they can affect the growth and migration of tumor cells [175]. OOC models have also been used to study the metastatic process of lung cancer, which is responsible for most lung cancer-related deaths. Researchers have developed a microfluidic model that mimics the blood vessel walls and allows for studying cancer cell invasion and migration through the endothelial barrier, creating a microfluidic platform that mimics the in vivo microenvironment of NSCL cells [176,177,178]. This microchip was used to test the cytotoxic effects of the drugs erlotinib (an EGFR tyrosine kinase inhibitor) and a novel anticancer agent NSC-750212 ([1-[(chlorophenyl)methyl]indol-3-yl]methanol). A tumor lung-on-chip model was created by Yang et al. [179] to test the effects of the anticancer drug—gefitinib (a tyrosine kinase inhibitor) on a co-culture of NSCL cancer cells, lung fibroblasts, and human endothelial cells. Also, using an organ tumor model, Khalid et al. [180] tested the effect of doxorubicin (DOX) and docetaxel (an antimitotic drug) on the apoptosis process in lung tumors. In another experiment, using microfluidic technology, co-cultures between human lung carcinoma cells and human amniotic membrane-derived mesenchymal stem cells (AMMSC) were performed, observing how the applied therapy influences the process by the development of tumor spheroids [181,182].

These tumor organ chip platforms can be used to develop and apply personalized therapy in different forms of cancer. Also, these devices allow the analysis of how the tumor microenvironment can influence the delivery of therapeutic agents, thus facilitating the development of more efficient drug administration systems at the tumor site [183,184].

Tumor organ-on-chip technology can be used in lung cancer immunotherapy by providing a platform to study the interactions between tumor cells and the immune system effectors and the effects of different immunotherapeutic agents on the tumor microenvironment. Tumor organ-on-chip facilitates the evaluation of the impact of cytokine therapies, which boost the immune system’s ability to attack cancer cells, and the effects of CAR-T cell therapies, which genetically modify the patient’s immune cells to target cancer cells [185,186]. For example, tumor organ-on-chip models can test the efficacy of checkpoint inhibitors, which block the inhibitory signals of the tumor cells used to evade the immune system. One type of immunotherapy aims to improve the immune response against cancer cells by blocking programmed cell death protein 1 (PD-1), a protein that induces inhibition of the immune system during the formation of the PD-1/PD-L1 pathway by targeting PLD1 (programmed cell death ligand1), which is involved in tumor cell growth and survival. Tumor cells are known to have the ability to escape immune surveillance and continue to proliferate. Thus, in recent years in cancer therapy, targeting PD-1 and PD-L1 has led to obtaining immune checkpoint inhibitors, such as anti-PD-1 and anti-PD-L1 antibodies, to block the interaction between PD-1 on T cells and PD-L1 on cancer cells [187].

Recent studies have shown that these inhibitors may significantly increase the treatment benefit of advanced NSCLC, especially in patients with high levels of PD-L1 expression [188].

The use of tumor organ-on-chip technology in the study of lung cancer offers the advantage of recreating the complex microenvironment of lung tumors, including the extracellular matrix, blood vessels, immune cells, and other components. Also, these devices ensure the simulation of the behavior of immune cells in the tumor microenvironment and allow for the study of the mechanisms of interaction between immune cells and tumor cells. Studies using tumor organ-on-chip platforms facilitate drug screening by testing the effectiveness of different immunotherapeutic agents on patient-derived tumor cells and monitoring the response in real time. The data obtained provide valuable information on the patient’s responses, offering the possibility of personalized treatment approaches. With the help of microtechnology devices, the mechanisms underlying tumor-immune cell interactions can be investigated, including the expression of immune checkpoint proteins and their role in regulating immune responses to facilitate the development of new immunotherapeutic strategies.

OOCs have an essential role in evaluating the toxicity of immunotherapies on healthy cells within the chip, providing a controlled environment to assess the safety profiles of different treatment regimens.

New 3D culture and microfluidic technology approaches are needed to improve lung cancer therapy. Thus, by developing new and more sophisticated 3D culture models, it is possible to better understand lung tumor cells’ behavior and biology, leading to improved therapeutic strategies. Using 3D culture models and microfluidic technology to study drug resistance mechanisms in lung cancer helps identify new therapeutic targets and strategies to overcome this challenge. For the created models to apply to real clinical situations, a close collaboration between clinicians and researchers from different fields (medicine, biology, biochemistry, biotechnology) is necessary to obtain better patient results. The combination of PD-1/PD-L1 inhibitors and tumor organ-on-chip is promising for advancing lung cancer therapy (Table 4). The information presented in this review supports the evolving role of 3D cultures in better understanding various aspects of the tumor microenvironment and its impact on tumor progression, gene and protein expression, pro-oncogenic signaling pathways, and drug resistance. Also, 3D cultures are a promising platform for developing microfluidic technology, thus ensuring more exact screening of drugs, including those used in immunotherapy, targeted drug administration, and noninvasive monitoring. These advances can lead to more effective and personalized treatments for different pathologies, a better understanding of disease mechanisms, and improved patient outcomes.

## 4. Challenges and Future Directions

3D modeling of lung cancer is a promising field with the potential to improve our understanding of the disease, aid in diagnosis and treatment planning, and enhance medical education.

The future perspectives of 3D microfluidic technology in the field of lung cancer are promising and could have a significant impact on both diagnosis and treatment.

3D microfluidic technology can enhance the sensitivity and specificity of liquid biopsies, which can detect circulating tumor cells or tumor-derived genetic material in the bloodstream. This could be a noninvasive way to diagnose and monitor lung cancer progression.

Microfluidic devices can be used to create miniaturized models of lung tumors, allowing for rapid testing of different drugs and treatment strategies on patient-specific cancer cells. This can help identify the most effective treatment options and reduce the time and cost of drug development.

Also, 3D printing and organ-on-chip technology can assist surgeons in creating patient-specific models of the lung and tumor for preoperative planning. This can lead to more precise and minimally invasive surgeries, reducing complications and recovery times.

Integrating data from 3D microfluidic technology with other medical imaging modalities, such as CT scans and MRIs, can provide a more comprehensive view of the tumor and its surroundings.

It is important to note that, while the potential for 3D microfluidic technology in lung cancer diagnosis and treatment is vast, there are still challenges to overcome, including technical hurdles, cost considerations and regulatory approvals. Building and manipulating 3D models of the lung and its tumors require substantial computational power and specialized software. These resources can be expensive and may not be accessible to all healthcare institutions. Also, implementing 3D modeling into clinical practice can be challenging due to the need for workflow integration, training of medical professionals, and compliance with regulatory standards. Validating the accuracy of 3D models is challenging, as there may not always be a ground truth to compare against. Establishing reliable validation methodologies is crucial for clinical acceptance. Additionally, interdisciplinary collaboration between engineers, clinicians, and researchers will be crucial to realizing the full potential of these technologies in the fight against lung cancer.

In conclusion, 3D modeling of lung cancer holds great promise for personalized medicine, research, and education, but it faces several substantial challenges related to data, technology, validation, integration into clinical practice, and ethical considerations. Addressing these challenges will be essential for the field to reach its full potential in improving lung cancer diagnosis and treatment.

## Figures and Tables

**Table 1:** The specific applications of spheroid models in lung cancer research.

Spheroid Model Applications	The Objective of the Study	Refs.
Tumor biology	to analyze the cellular and molecular mechanisms involved in lung tumor formation, growth and progression	[70,71]
Drug discovery	to test the effectiveness of different new drugs on lung cancer cells	[48,73]
Treatment response	to predict the response of lung tumor cells to chemotherapy or radiation therapy and to evaluate the efficacy of other combination therapies in a more realistic environment	[77,78]
Metastasis	to study the process of lung cancer metastasis, including the migration and invasion of lung cancer cells into surrounding tissues	[79,80]
Angiogenesis	to study the formation of new blood vessels in lung tumors, critical for tumor growth and survival	[81]

**Table 2:** The applications of organoid models in lung cancer research.

Organoid Model Applications	The Objective of the Study	Refs.
Studying cancer biology	to study the underlying biology of lung cancer cells, such as how they grow, migrate and respond to stimuli	[91,92]
Drug testing	to test the efficacy of new drugs; organoids can be treated with different drug compounds and their effects on disease progression or cellular function can be analyzed	[100]
Drug delivery optimization	can be used to evaluate drug delivery methods and test different drug formulations and delivery systems to determine which are most effective at reaching and targeting specific cells or tissues	[101]
Screening for drug resistance	can be used to study drug-resistance mechanisms in diseases such as cancer	[102]
Target validation	can assist in validating drug targets by assessing the impact of genetic modifications or gene editing on disease phenotypes and drug responses	[103]
Personalized medicine	by testing drugs on organoids derived from a patient’s own cells, clinicians can identify the most effective treatment options while minimizing potential side effects; can help to identify specific genetic mutations or other characteristics that drive a patient’s cancer and suggest personalized treatment strategies	[104,105]

**Table 3:** Types of organ-on-chip models used in research and medicine.

Type of Organs-on-Chip	Application	Refs.
Liver-on-chip model	mimics the complex architecture and function of the liver and is used to study liver diseases, drug toxicity, and drug metabolism	[143,144]
Heart-on-chip model	mimics the heart’s contractile activity and electrical properties and is used to study cardiovascular diseases and the effects of drugs on cardiac tissue	[145,146]
Lung-on-chip model	mimics the air-blood barrier of the human lung and is used to study respiratory diseases and the effects of toxins on lung tissue	[147,148,149]
Kidney-on-chip model	mimics the structure and function of the nephron and is used to study kidney diseases and drug toxicity	[150,151]
Intestine-on-chip model	mimics the structure and function of the intestinal epithelium and is used to study intestinal diseases and drug absorption	[152,153]
Colon-on-a-chip model	mimics the human colonic mucus layer structure and function to analyze the role of mucus in ulcerative colitis and cancer	[154]
Brain-on-chip model	mimics the structure and function of the blood-brain barrier and is used to study neurological diseases, drug delivery to the brain, and treatment effects	[155]

**Table 4:** Potential practical applications of tumor organ-on-chip tests for improving lung cancer treatment.

Tumor-Organ-on-Chip Test	Studied Effect	Practical Potential Applications	Refs.
Testing the physiological conditions in a realistic tumor microenvironment	Study of mechanisms of tumor development and effects of PD-1/PD-L1 blockade on immune cell function	Simulation of physiological conditions to analyze the infiltration process of immune cells and the established interactions with tumor cells	[189]
Testing the efficacy of PD-1/PD-L1 inhibitors	Evaluating the effects induced by combining immunotherapy with other types of drugs and identifying potential synergistic treatments	Finding new drugs and developing more effective therapeutic strategies	[190]
Investigation of the underlying mechanisms of therapy resistance	Understanding the mechanisms of resistance and targeting the molecules responsible for establishing resistance to a specific type of treatment	Development of strategies to overcome the resistance to therapy	[191,192]
Using the patient’s cells in tumor organ-on-chip to achieve personalized testing of different treatment strategies	Identifying the most effective PD-1/PD-L1 inhibitor may guide treatment decisions based on the specific characteristics of an individual’s tumor	Personalized medicine	[193]
